# Smartphone image dataset for radish plant leaf disease classification from Bangladesh

**DOI:** 10.1016/j.dib.2024.111263

**Published:** 2024-12-27

**Authors:** Mahamudul Hasan, Raiyan Gani, Mohammad Rifat Ahmmad Rashid, Maherun Nessa Isty, Raka Kamara, Taslima Khan Tarin

**Affiliations:** Department of Computer Science and Engineering, East West University, Aftabnagar, Dhaka, Bangladesh

**Keywords:** Disease identification, Dataset collection, Image analysis, Leaf diseases, Agricultural challenges, Radish plant leaf disease

## Abstract

Radishes, which are common root vegetables, are rich in vitamins and minerals, and contain low calories. This vegetable is known for its rapid growth. Nevertheless, the variety of leaf diseases where leaves get affected by various bacterial and fungal diseases can hinder the healthy growth of radish. Furthermore, there is a high risk of inaccurate identification of diseases if the farmers try to use traditional methods in recognizing these diseases. With the purpose of precise identification of radish leaf diseases for the finest growth of this vegetable, total of 2801 images of the radish leaves are collected from vegetable field in Bangladesh. The collected dataset includes comprehensive images of healthy leaves as well as four types of leaf affected by various diseases such as Black Leaf Spot, Downey Mildew, Flea Beetle and Mosaic. Utilizing this robust dataset, deep learning models can be trained to identify the leaf diseases which helps to detect the diseases in order to reduce the harm of the cultivation of radish. By identifying the diseases on radish leaves accurat-ely and maintaining healthy production of radish, this dataset contributes to the broader sustainability in the agricultural sector.

Specifications Table

**Table:** 

Subject	Plant Pathology branch of Agriculture
Specific subject area	Radish Plant Leaf Disease Identification and Classification.
Type of data	Raw Images Dataset in 700×700 jpg format.
Data collection	In order to address advancement in the agricultural sectors, we are able to organized a well-structured radish plant leaf disease where the dataset contains overall 2801 images of radish plant leaves. The dataset is divided into five classes where four classes hold different types of radish plant leaf diseases and one class contains the healthy plant leaves in order to compare the unhealthy or the affected leaves with the healthy leaves. The images were captured with an android smartphone Realme 9 5G. The images were captured in a well-organized laboratory area so that the original contrast of each image is sustained. Furthermore, the pictures were taken in multiple angles so that researchers can have the overall glance of a particular leaf. The dataset will play an important role for classifying the radish plant healthy and unhealthy leaves.
Data source location	1. Vegetable field of Kathalkandi, Nasirnagar, Brahmanbaria, Bangladesh (latitude: 24.1915°, longitude: 91.1826°)
Data accessibility	Repository name: Mendeley Data Data identification number: 10.17632/s973cz2jcd.1 Direct URL to data: https://data.mendeley.com/datasets/s973cz2jcd/1

## 1 Value of the Data

• The dataset containing several classes of radish leaves where each class clearly representing the unhealthy leaf as well as healthy leaf. All the images are captured with high resolution that ensuing the high-quality of leaves images, helps to recognize the pattens of diseases.
• The dataset presents practical information about the diseases that lessen the production of Radish where scientists can utilize this dataset to identify a way that will be useful in increasing the productivity of radish cultivation as well as preventing leaf diseases.
• The dataset is shared publicly which is valuable for conducting open research. This dataset will encourage scientists and researchers to do more research by training machine learning and deep learning models to identify and classify the diseases automatically and help in agriculture sectors by developing real-time diseases diagnoses system though mobile or web applications.
• This dataset will also be beneficial for the country's vegetable growth through early and accurate detection of leaf diseases which guides the farmers to maintain yield and apply appropriate pesticides that brings great profit in the economy as well.

## 2 Background

Radish is often known as a balanced dietary vegetable which contributes to the food stability mostly cultivated in Southeast Asian countries during the cool season. Radish leaf diseases can significantly reduce the growth of radish and its production. Typically, it is challenging for the farmer to detect diseases on radish leaf by using older methodology. Hence, improvement in detecting these leaf diseases through advanced technologies is essential to identify the diseases accurately. Furthermore, many researchers are working hard to identify the plant leaf diseases [1] in order to identify the early detection of the harmful diseases so that farmers can take necessary steps to prevent them. Moreover, due to the lack of technological support for the farmers, many countries are suffering for the low food cultivation rate which also affect their daily vegetable need and economy. In order to have an advance technology to prevent the leaf disease identification issue, we need a well distributed classified dataset to apply advance technology. Deep learning models are playing an important role in classifying the unhealthy leaves. Many researchers [2] worked in the healthy and diseased rice leaves with total 2586 images to classify rice plant leaf diseases using various convolutional neural network models, with the MobileNet model achieving the highest accuracy of 90%. Few researchers [3] already published a dataset on the papaya leaf plant where they are have discovered five leaf diseases of papaya leaf which will help the researcher to train the deep learning models and markdown an advancement in the computer vision segment. Another dataset [4] comprising over 9000 high-quality soybean images aids in soybean leaf disease classification which will play crucial role in for identifying the soybean diseases. The integration of deep learning in plant disease detection not only revolutionizes smart agriculture but also contributes significantly to sustainable farming practices, food security, and early disease management in crops. For this reason, a robust dataset on radish leaf diseases is important so that the models can be trained with high performance to find the diseases precisely.

## 3 Data Description

**Fig. 1:**
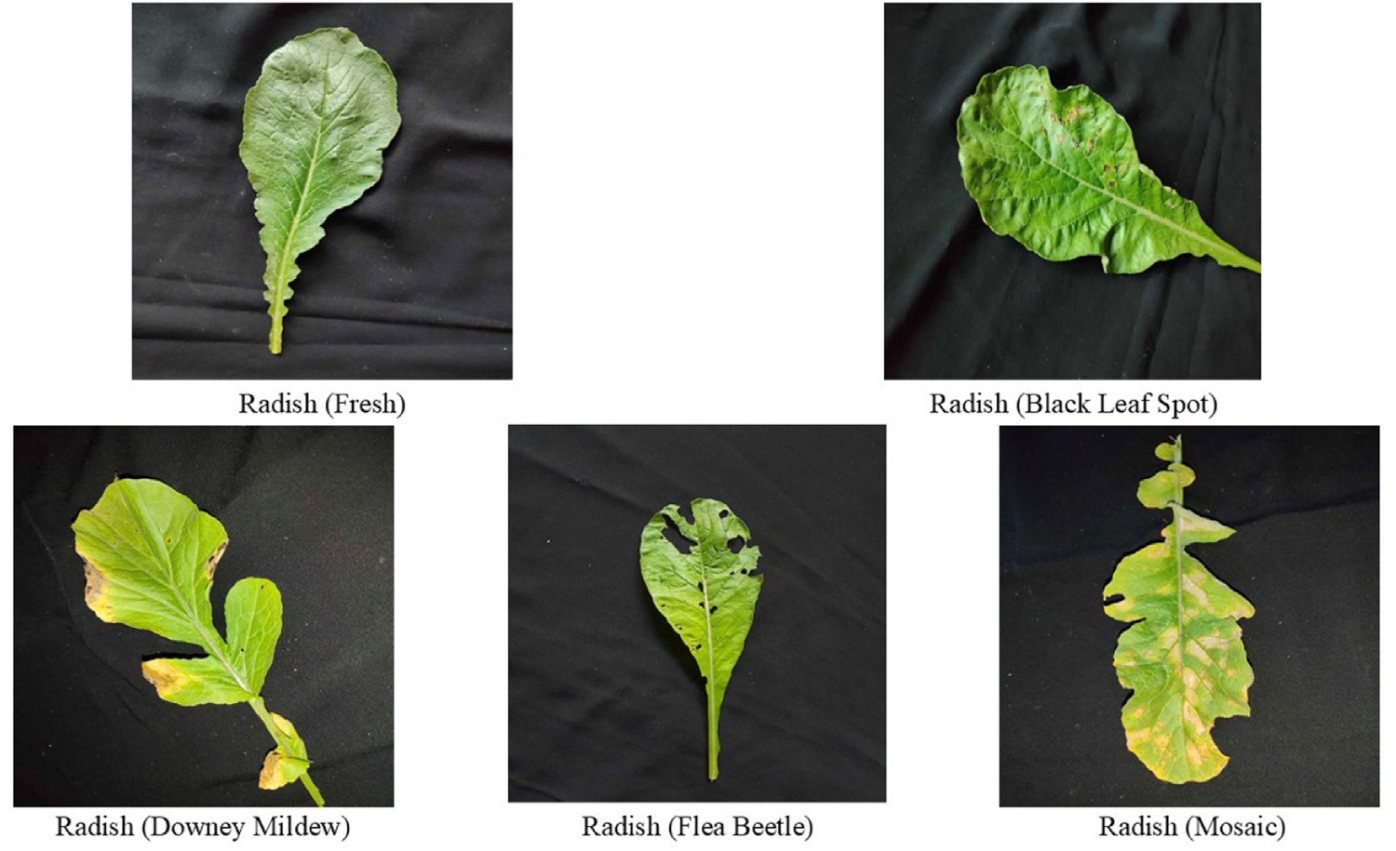
Images of Radish leaf from every class.

Our dataset [5] provides 2801 images of four different classes of diseased leaves and one class of fresh leaves collected from the field of Kathalkandi, Nasirnagar, Brahmanbaria, Bangladesh. Fig. 1 shows the five distinct classes of Radish such as Fresh leaf, Black leaf spot, Downy mildew, Flea beetle, and Mosaic virus. The images are organized based on the fresh and diseased class types which will help researchers to have a clear insight of the dataset. This dataset can be useful to researchers for understanding, and analysing the most devastating Radish leaf diseases that hinder crop yield in Bangladesh or countries with similar climates.

### 3.1 Black leaf spot

**Fig. 2:**
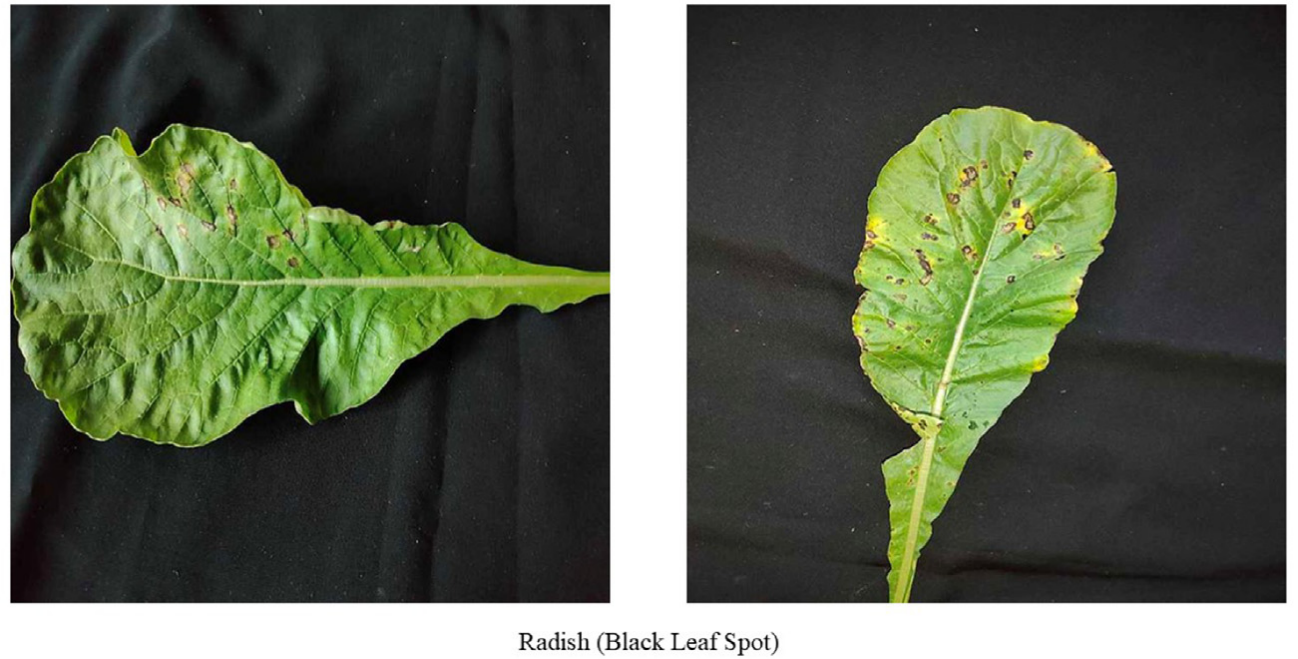
Images of radish leaf with black leaf spot disease.

Fig. 2 shows the Radish leaf with Black leaf spot disease. The symptoms may appear variously depending on the agent. Normally water-soaked black lesions with yellowish ring spots, V-shaped spots are visible on radish leaf due to the attack of Xanthomonas campestris pv. Armoraciae pathogen [6,7].

### 3.2 Downy mildew

**Fig. 3:**
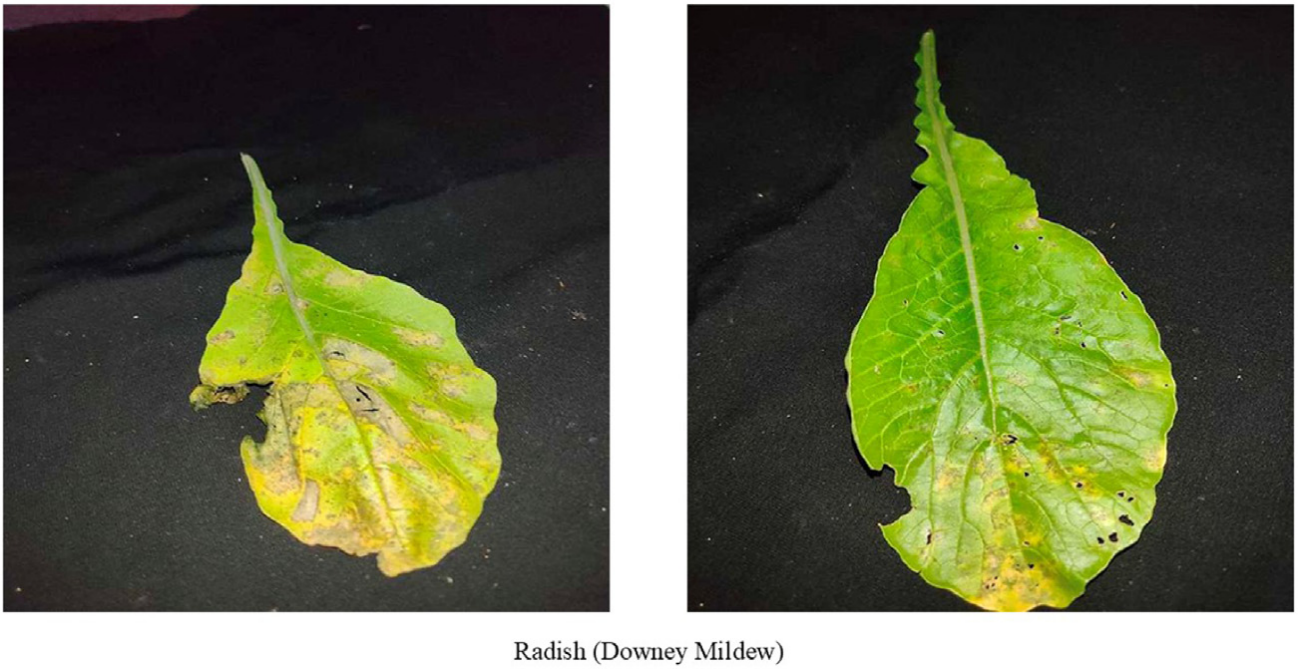
Images of radish leaf with Downy mildew disease.

Peronospora parasitica fungus causes Downy mildew disease shown in Fig. 3, which is responsible for great damage in radish cultivation worldwide. The leaves appear with brown or yellow lesions on the top and white or grey mycelium on the bottom [8]. This disease gradually leads to leaf death and hampers radish production.

### 3.3 Mosaic virus

**Fig. 4:**
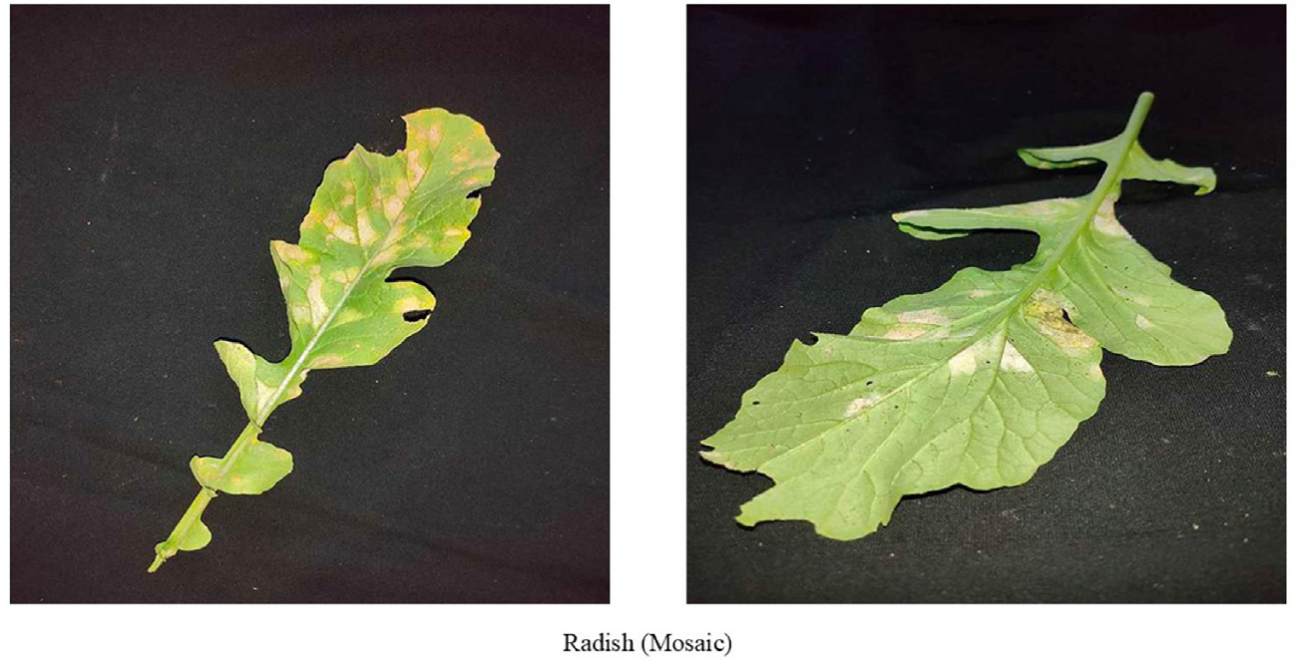
Images of radish leaf with Mosaic virus disease.

Mosaic virus is a great threat to almost all vegetables. From Fig. 4, we can see when the Mosaic virus attacks radish plant green leaves gradually become yellow. Other common symptoms [9] are mosaic patterns and chlorosis on the leaves. Leaf curbing is also seen when this disease attacks plants. Early detection is crucial to reduce the damage done by the Mosaic virus.

### 3.4 Radish flea beetle

**Fig. 5:**
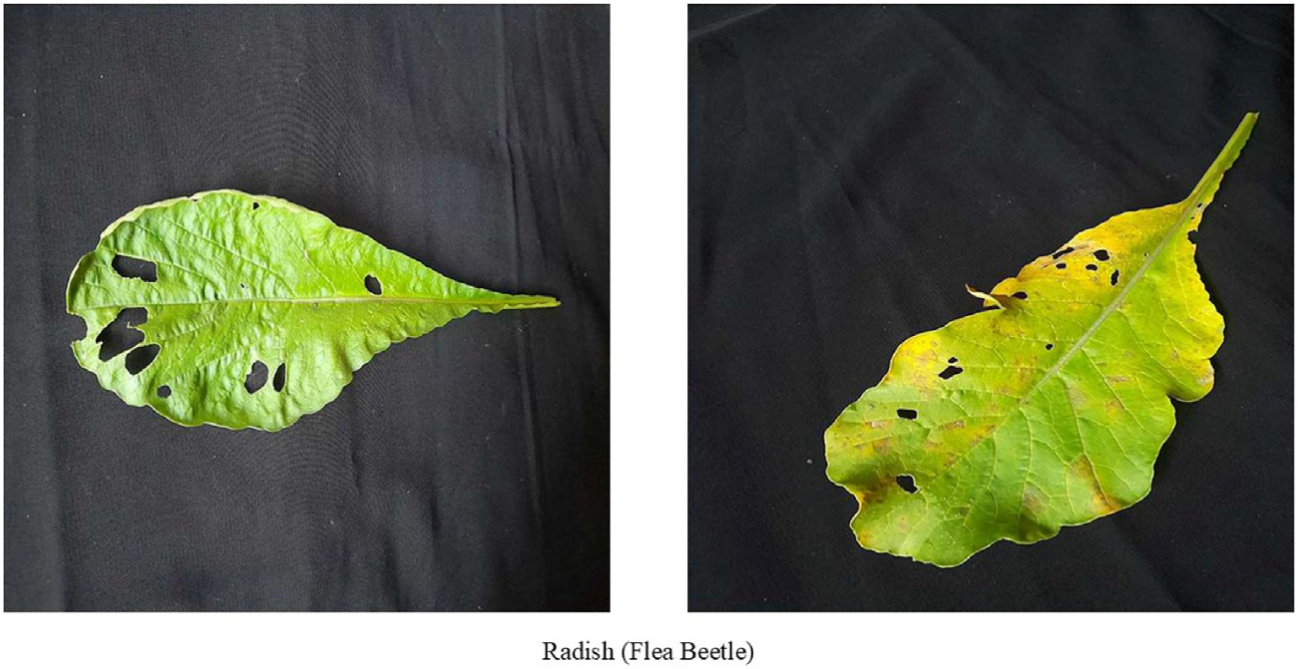
Images of radish leaf with flea beetle disease.

Radish flea beetle disease is one of the most common diseases that reduce radish crop production. From Fig. 5 illustrates that mature flea beetle creates round and tiny holes [10] on leaves. The holes destroy the green colours of leaves. If farmers delay identifying the disease, flea beetles feed on leaves and kill young plants. On mature plants holes and scars created by this pest, reduce the crop worth.

### 3.5 Fresh leaf

**Fig. 6:**
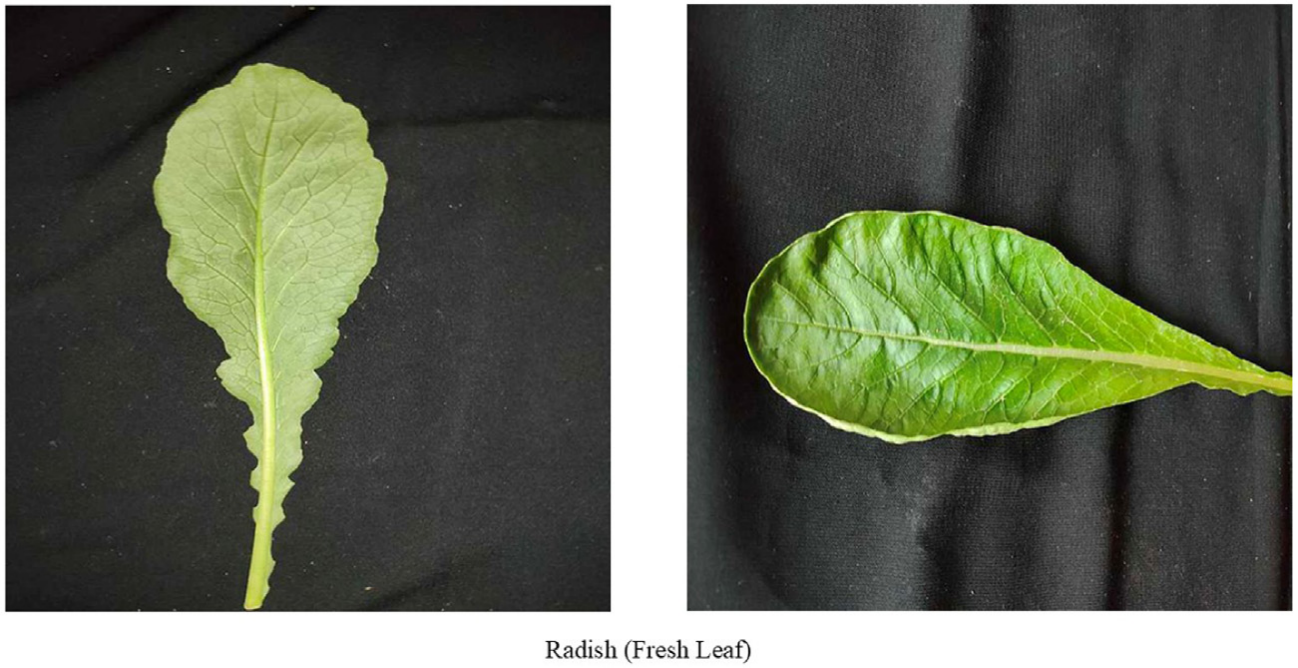
Images of fresh leaf of radish.

A fresh leaf of Radish shown in Fig. 6 has a smooth surface along with a sharp and little wrinkled texture. The colour of a fresh radish leaf is vibrant green and the shape is lobed and can appear in various sizes.

**Fig. 7:**
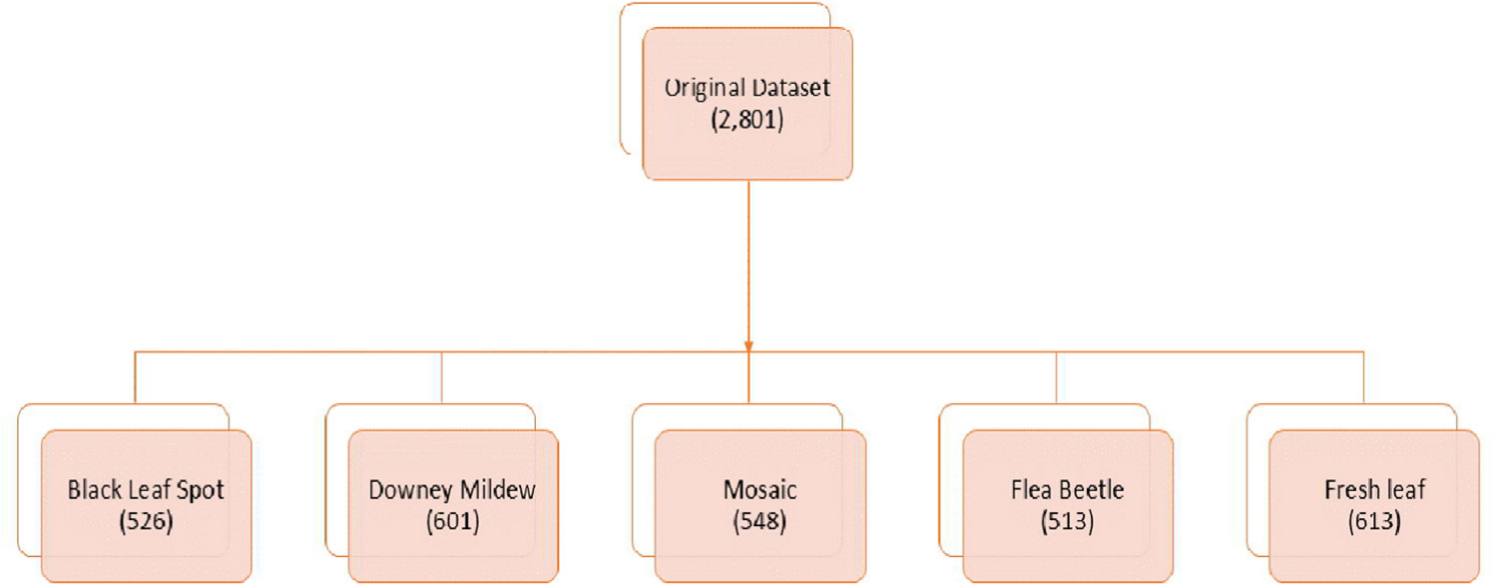
Dataset class folder information.

Table 1 shows that the dataset presents the images in the main directory labelled as “Radish” which has five subdirectories. For uniformity, all the images were resized. To prevent bias and achieve better accuracy while training machine or deep learning models the dataset was carefully designed so that no minority class is present. The distribution of the dataset shown in Table 1 indicates that it is finely distributed. To help find any image from a particular class easily a folder structure is shown in Fig. 7.

**Table 1:** Dataset sample distribution.

Original Dataset				
Name of the Vegetable	Class	Category	No. of Images	Folder Name
Radish	1	Black leaf spot	526	Black leaf spot
	2	Downy mildew	601	Downey mildew
	3	Mosaic virus	548	Mosaic virus
	4	Radish flea beetle	513	Radish flea beetle
	5	Fresh leaf	613	Fresh leaf

## 4 Experimental Design, Materials and Methods

### 4.1 Dataset collection and preparation

**Fig. 8:**
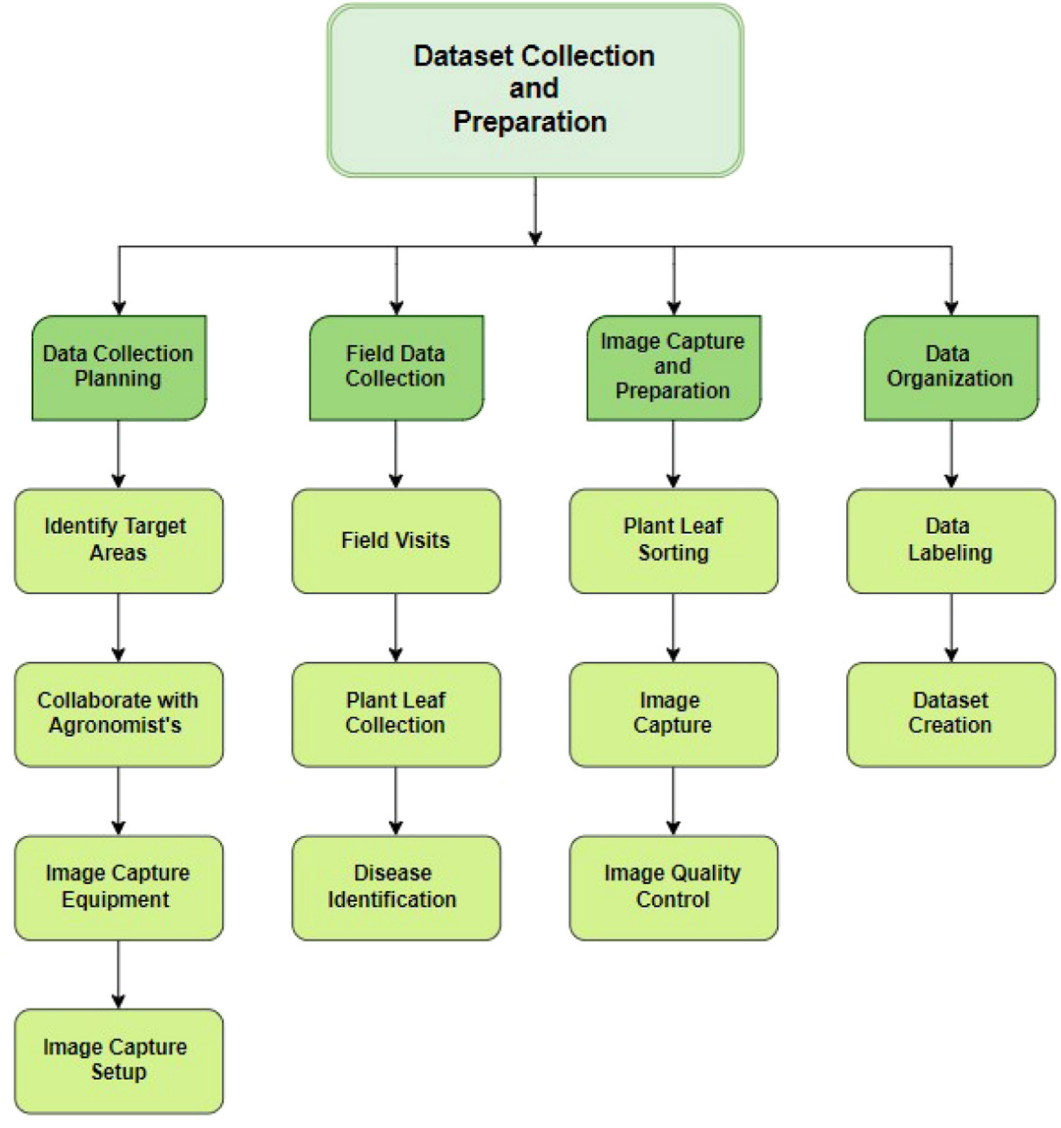
Overall working procedure.

A fundamental component in plant disease diagnostics is the acquisition of data of the highest caliber. Fig. 8 illustrates the overall working procedure of our research. To construct a robust and comprehensive dataset, we undertook extensive field surveys across varied agronomic landscapes in Kathalkandi, Nasirnagar, and Brahmanbaria, Bangladesh.

Our research involved an exhaustive analysis of an extensive array of leaf pathologies, providing a profound understanding of each specific disease. By closely collaborating with local agronomists, we meticulously pinpointed vegetable plots and amassed detailed insights. This approach allowed us to achieve very accurate diagnoses and classifications of affected plant tissues. By using advanced diagnostic methods and drawing on local expertise, we ensured a high level of precision in assessing these rare and often difficult-to-diagnose diseases. This careful process not only improved our understanding but also strengthened our ability to tackle and manage these unique agricultural problems more effectively.

In the image acquisition phase, we used a Realme 9 5G smartphone, capturing each leaf from six distinct perspectives under rigorously controlled lighting conditions. Table 2 illustrates the overall camera specifications of Realme 9 5G smartphone. A custom-designed laboratory environment in order to preventing the over sunlight was precisely organized to maintain exact color fidelity and mitigate the risk of oversaturation, thereby optimizing diagnostic accuracy and reliability.

**Table 2:** Image captured smartphone device specification.

Device Name	Camera Resolution
Realme 9 5G	48MP AI Triple Camera, 48MP UHD Main Camera, Aperture F1.8, FOV 79.8°, Equivalent focal length 25.4mm, 6P lens, Sensor dimensions 1/2′, 4cm Macro lens, 2 million pixels, Aperture F2.4, FOV 88.8°, Focus distance 4cm, Portrait lens, 2 million pixels, Aperture F2.4, FOV 88.8°, Equivalent focal length 21.8mm, 3P lens

We assiduously collected and systematically cataloged leave from the vegetable fields. We have selected 8 distinct leaves for Fresh class, 13 distinct leaves for Black leaf spot, 4 distinct leaves for Downey mildew, 4 distinct leaves for Mosaic virus, and 12 distinct leaves for Flea beetle. The number of distinct leaves were selected by considering the leaf quality and availability in the selected crop field. Furthermore, for each leaf, we have captured at least 15 to 20 images per orientation and we tried to cover the overall leaf properties by manually rotating the leaf position. With the following vegetable species represented in our dataset, we implemented exacting classification procedures to prevent misidentification. Accurate grouping, aligning leaves with their appropriate category, was critical to circumventing potential errors and discrepancies. The diseased leave was thoroughly classified to uphold diagnostic precision. To minimize visual noise, we utilized a black velvet backdrop, which enhanced the camera's capacity to achieve precise focus and capture the nuanced details of disease manifestations with exceptional clarity and improved our ability to analyze and understand the subtle signs of diseases.

## Limitations

While following all the research procedures, we have encountered many difficulties and overcome them it a basic solution. The limitations of our research work are mentioned below;

• **Data Collection:** As we have only visited the vegetable fields of Brahmanbaria, Chittagong, Bangladesh. Thus, it was quite hard for us to identify the radish crop fields and collect all the images according to the mentioned classes. In order to have a good understanding with the radish crop field, we took the help of the local farmers in Chatalpar where they have helped us to mark down the radish fields that were quite good for data collection. As we have understood that it is important to have a clear understanding about each disease. Otherwise, it will be very difficult to categorize the dataset.
• **Image Capturing:** While capturing the images, the sunlight was over saturating the images quality which may add additional noise to the images and the disease spots will be difficult to identify. To overcome the situation, we have managed to create a controlled environment where we have controlled the sunlight and managed to click the images into six different angles.
• **Data Augmentation:** We have not augmented our original dataset. We have also trained the deep learning models with the actual dataset. Our dataset is well balanced. However, if we enlarge the dataset amount it may help the deep learning models to learn model and able to predict the real-world data more accurately.
• **Real time Disease Detection:** As we have trained the deep learning models, we haven't integrated the trained models into a mobile application. Thus, further research can be done by introducing an application in order to fund the radish real time leaf disease detection which will be helpful for the farmer to identify the actual disease and take the necessary cures**.**

## Ethics Statement

The authors adhere to the journal's ethical guidelines and confirm that this research does not involve humans, animals, or data obtained from social media. The datasets utilized in the study are publicly accessible, and appropriate citation protocols should be followed when utilizing these datasets.

## CRediT authorship contribution statement

**Mahamudul Hasan:** Conceptualization, Methodology, Supervision, Visualization, Project administration, Validation. **Raiyan Gani:** Investigation, Methodology, Supervision, Writing – original draft, Writing – review & editing. **Mohammad Rifat Ahmmad Rashid:** Conceptualization, Methodology, Supervision, Visualization, Project administration, Validation. **Maherun Nessa Isty:** Investigation, Writing – original draft, Writing – review & editing. **Raka Kamara:** Investigation, Methodology, Writing – original draft, Writing – review & editing. **Taslima Khan Tarin:** Investigation, Writing – original draft, Writing – review & editing.

## Data Availability

Mendeley DataImage Dataset for Radish Plant Leaf Disease Detection and Freshness Assessment from Bangladesh (Original data).
